# *Hypomyces pseudolactifluorum* sp. nov. (Hypocreales: Hypocreaceae) on *Russula* sp. from Yunnan, PR China

**DOI:** 10.3897/BDJ.8.e53490

**Published:** 2020-09-28

**Authors:** Feng-ming Yu, Ruvishika S. Jayawardena, Jianwei Liu, Kevin D. Hyde, Qi Zhao

**Affiliations:** 1 Key Laboratory of Economic Plants and Biotechnology, Kunming Institute of Botany, Chinese Academy of Sciences, Kunming, China Key Laboratory of Economic Plants and Biotechnology, Kunming Institute of Botany, Chinese Academy of Sciences Kunming China; 2 Center of Excellence in Fungal Research, Mae Fah Luang University, Chiangrai, Thailand Center of Excellence in Fungal Research, Mae Fah Luang University Chiangrai Thailand; 3 Institute of Plant Health, Zhongkai University of Agriculture and Engineering, Guangzhou, China Institute of Plant Health, Zhongkai University of Agriculture and Engineering Guangzhou China; 4 Institute of Applied Fungi, Southwest Forestry University, Kunming, China Institute of Applied Fungi, Southwest Forestry University Kunming China

**Keywords:** Mycoparasite, species diversity, muti-gene phylogeny

## Abstract

**Background:**

*Hypomyces* is a large genus of fungicolous fungi, parasitising the fruiting bodies of Agaricales, Boletales, Helotiales, Pezizales and Polyporales. *Hypomyces* currently comprises of 147 species widely distributed in Australia, China, France, Germany, Italy, Japan, North America, Sri Lanka, Thailand and UK. Amongst them, 28 species have been recorded in China.

**New information:**

*Hypomyces pseudolactifluorum* sp. nov., growing on the fruiting bodies of Russula sp. in subsect. Lactarioideae and collected from Yunnan, China, is described with illustrations and molecular phylogenetic data (combined ITS, LSU, *TEF1*-*α* and RPB2 sequence dataset). The new species is characterised by semi-immersed to immersed perithecia and fusiform, apiculate and verrucose ascospores. We also review the species diversity of the genus *Hypomyces* in China.

## Introduction

Fungicolous fungi are a large and diverse ecological group, currently containing more than 1500 taxa distributed in many lineages across the fungal kingdom (Põldmaa 2011, Sun et al. 2019a). *Hypomyces* (Fr.) Tul. & C. Tul. is an important genus of fungicolous fungi and placed in the family Hypocreaceae (Hypocreales, Sordariomycetes, Ascomycota) (Hyde et al. 2020). *Hypomyces* was originally introduced as a subgenus of *Hypocrea* Fr. (Fries 1825) and then Tulasne and Tulasne (1860) revised it to a genus and designated *H. lactifluorum* (Schwein.) Tul. & C. Tul. from the USA as its type. *Hypomyces* parasitises the fruiting bodies of Agaricales, Boletales, Helotiales, Pezizales and Polyporales (Rossman et al. 1999, Tamm and Põldmaa 2013, Sun et al. 2019a). *Hypomyces* is characterised by: superficial or immersed, spherical to ovate, pyriform, papillate and yellow, orange, tawny red or green perithecia in a subiculum; 8-spored, subcylindrical to cylindrical and with a thickened apical asci; and ellipsoid, lanceolate, fusiform to navicular, 0-1-septate or rarely 3-septate, hyaline, spinulose or verrucose and smooth-walled ascospores (Rossman et al. 1999, Zeng and Zhuang 2015). Its allied genera include *Cladobotryum* Nees, *Mycogone* Link, *Sepedonium* Link and *Stephanoma* Wallr (Wijayawardene et al. 2017) and its asexual morphs are *Acremonium*-, *Dactylaria*-, *Papulaspora*-, *Trichothecium*- or *Verticillium*-like (Jaklitsch et al. 2006, Hyde et al. 2020). *Hypomyces* currently comprises of 147 species in Species Fungorum (http://www.speciesfungorum.org/, accessed in April 2020) and is widely distributed in Australia, China, France, Germany, Italy, Japan, North America, Sri Lanka, Thailand and UK (Zhuang et al. 2012, Rossman et al. 2013, Zeng and Zhuang 2016, Zare and Gams 2016, Lechat et al. 2017, Wei and Kirschner 2017, Sun et al. 2019a, Sun et al. 2019b, Zeng and Zhuang 2019). Amongst them, 28 species have been reported in China (Table 1).

Fungicolous fungi play important roles in the processes of the growths and degradations of their hosts. With the rapid development of mushroom industries, the fungicolous fungi on mushrooms have received more and more attention (Hyde et al. 2019). In this paper, we introduce a new member of fungicolous fungi, *Hypomyces pseudolactifluorum* sp. nov., on the fruiting bodies of *Russula* sp., collected from Yunnan Province, China. At the same time, we review the species diversity of the genus *Hypomyces* in China.

## Materials and methods

**Collections and Morphology**

*Hypomyces* specimens, including their host mushrooms, were collected in an evergreen broad-leaved forest in Baihualing, Baoshan, Yunnan Province, China. The specimens, as well as collected host mushrooms, were placed on a piece of aluminium foil at first, then rolled the paper into a cylinder, twisted at the ends for sealing and lastly taken back to the laboratory for study (McKnight and McKnight 1997). Colour codes were recorded following those of Kornerup and Wanscher (1978). A Nikon Coolpix P510 camera was used to take photos in the wild. Dried specimens were observed and photographed using an Olympus SZ61 stereomicroscope and a Nikon ECLIPSE Ni compound microscope fitted with a Canon EOS 600D digital camera. Measurements were made using the Tarosoft® Image Frame Work programme v.0.9.7. The colour change of the perithecial wall was tested using 5% potassium hydroxide (KOH). Type specimens are deposited at the Herbarium of Mae Fah Luang University, Thailand (MFLU) and the Herbarium of Cryptogams Kunming Institute of Botany, Chinese Academy of Sciences, PR China (HKAS).

**DNA extraction, PCR amplification and sequencing**

The genomic DNA was extracted from the dried materials using the CTAB method (Doyle 1987). Tissues from the ascocarps of parasitic fungi and fruiting bodies of the host mushrooms were used to extract DNA, respectively. Primer pairs ITS1F/ITS4 (White et al. 1990), LR0R/LR5 (Rehner and Samuels 1994, Vilgalys and Hester 1990), *TEF1*-*α* 983f/*TEF1*-*α* 2218r (Carbone and Kohn 1999, Rehner and Buckley 2005) and RPB2-5f/RPB2-7cR (Liu et al. 1999) were used for amplification of the ITS, LSU, *TEF1*-*α* and RPB2 gene regions.

PCR was performed in a 25 μl reaction volume: 12.5 μl Taq PCR Master Mix (Abmgood, Richmond, BC, Canada), 1 μl forward primer, 1 μl reverse primer, 1 μl DNA template and 9.5 μl ddH_2_O. For ITS and LSU, PCR reaction conditions are: 8 min at 94ºC, followed by 30 s at 94ºC, 30 s at 52ºC and 1 min at 72ºC for 35 cycles and a final extension of 10 min at 72ºC. PCR reaction conditions of *TEF1*-*α* and RPB2 are: 8 min at 94ºC, followed by 1 min at 95ºC, 45 s at 59ºC for RPB2/55ºC for *TEF1*-*α* and 1 min at 72ºC for 35 cycles and a final extension of 10 min at 72ºC. The PCR products were detected using agarose gel electrophoresis and, in the gel documentation system, clear bands were observed. Sequencing was performed by Sangon Biotech (Shanghai) Co. Ltd., PR China; partial impure products were purified using the Cycle-pure-kit (Omega, America) and then cloned into pClone007 Simple vector (TSV-007S from Beijing TsingKe Biotech). Twenty clones of PCR products of each gene were sequenced using the universal primer pairs M13-47/M13-48.

**Sequence alignment and phylogenetic analyses**

**The parasitic fungus**: ***Hypomyces pseudolactifluorum*** **sp. nov.**

Molecular phylogenetic trees were constructed using our sequencing results of *H. pseudolactifluorum* sp. nov. and the voucher sequences of their allies obtained from NCBI GenBank (Table 2). Two species of *Trichoderma*, *T. hamatum* (DAOM 167057) and *T. viride* (CBS 119325) were used as outgroup taxa. All sequences were assembled and aligned using MAFFT v6.8 (Katoh et al. 2005) and manually edited via BioEdit version 7.0.9 (Hall 1999). Four sequence matrices of ITS, LSU, *TEF1-α* and RPB2 genes, respectively, were compiled. The optimal substitution model for each gene dataset was determined using jModelTest2 under the Akaike information criterion (AIC) (Darriba et al. 2012). The results indicated that the GTR+I+G model (-lnL = 8658.2624) is optimal for the ITS dataset, as well as the TIM1+I+G model (-lnL = 4392.5417) for LSU, the TrN+I+G model (-lnL = 5751.4959) for *TEF1*-*α* and the model SYM+I+G (-lnL = 6419.6669) for RPB2, respectively. Using the aligned sequence matrices, a combined gene sequence dataset (ITS, LSU, *TEF1-α* and RPB2, orderly) was assembled and aligned and was finally deposited in TreeBASE database (http://purl.org/phylo/treebase/phylows/study/TB2:S26593?x-access-code=152eadfc2292343af7627cfad5c2946c&format=html).

Maximum Likelihood (ML) analysis was performed using IQ-Tree (Nguyen et al. 2014, Chernomor et al. 2016) with the computing models listed above and a bootstrap test of 1000 replicates. Bayesian Inference (BI) analysis was carried out using MrBayes v3.2.6 (Ronquist et al. 2012). The TIM1 and TrN substitution models were replaced by the GTR model (Huelsenbeck and Rannala 2004). Four simultaneous Markov Chain Monte Carlo (MCMC) chains were run for random trees of 10,000,000 generations and were sampled by every 100 generations. The computing was stopped when the standard deviation of the split frequencies fell below 0.01 and ESS values > 200. Subsequently, phylogenetic trees were summarised and posterior probabilities (PP) were performed using MCMC by discarding the first 25% generations as “burn-in” (Huelsenbeck and Ronquist 2001). Gaps were treated as missing data. Phylogenetic trees were viewed in FigTree v.1.4.2 (http://tree.bio.ed.ac.uk/software/figtree).

**The host mushroom: *Russula* sp.**

Voucher sequences (ITS gene) for phylogenetic analyses of the host mushroom and its allies were obtained from our sequencing results and GenBank databases (Li et al. 2020) (Table 3). Five species of Russula subg. Compactae, *R. acrifolia, R*. *adusta, R*. *eccentrica, R*. *nigricans* and *R. subnigricans* were selected as the outgroup taxa. Sequence alignment and phylogenetic analyses followed those of the parasitic fungus above. ML analysis was performed using IQ-Tree with TVM+I+G model (-lnL = 5298.7964) (Nguyen et al. 2014, Chernomor et al. 2016). The ITS sequence matrix of the host mushroom and its allies were deposited in the TreeBASE database (http://purl.org/phylo/treebase/phylows/study/TB2:S26693?x-access-code=2e445b17aebe1f93266051a8920ae62f&format=html).

## Taxon treatments

### Hypomyces pseudolactifluorum

F. M. Yu, Q. Zhao & K. D. Hyde sp. nov.

1119A7CE-9897-55CC-94D7-5CF3C180F567

#### Materials

- **Type status:** Holotype. **Occurrence:** catalogNumber: MFLU 20-0265; recordedBy: Jian-Wei Liu; lifeStage: Telemorph; **Taxon:** scientificName: Hypomyces pseudolactifluorum; **Location:** country: China; stateProvince: Yunnan; locality: Baoshan, Longyang, Baihualing; verbatimElevation: 2094m; locationRemarks: label transliteration: "Yunnan, Baoshan, Longyang, Baihualing, on Russula sp., 20 July 2018, Jian-Wei Liu; [云南保山百花岭 2094 m, 2018.07.20, 刘建伟]; verbatimCoordinates: 25°17.931’N, 98°47.0718’E; decimalLatitude: 25.2989; decimalLongitude: 98.7845; georeferenceProtocol: label; **Identification:** identifiedBy: Feng-Ming Yu; dateIdentified: 2019
- **Type status:** Paratype. **Occurrence:** catalogNumber: MFLU 20-0266; recordedBy: Jian-Wei Liu; lifeStage: Telemorph; **Taxon:** scientificName: Hypomyces pseudolactifluorum; **Location:** country: China; stateProvince: Yunnan; locality: Baoshan, Longyang, Baihualing; verbatimElevation: 2094m; locationRemarks: label transliteration: "Yunnan, Baoshan, Longyang, Baihualing, on Russula sp., 20 July 2018, Jian-Wei Liu; [云南保山百花岭 2094 m, 2018.07.20, 刘建伟]; verbatimCoordinates: 25°17.931’N, 98°47.0718’E; decimalLatitude: 25.2989; decimalLongitude: 98.7845; georeferenceProtocol: label; **Identification:** identifiedBy: Feng-Ming Yu; dateIdentified: 2019
- **Type status:** Isotype. **Occurrence:** catalogNumber: HKAS 107300; recordedBy: Jian-Wei Liu; lifeStage: Telemorph; **Taxon:** scientificName: Hypomyces pseudolactifluorum; **Location:** country: China; stateProvince: Yunnan; locality: Baoshan, Longyang, Baihualing; verbatimElevation: 2094m; locationRemarks: label transliteration: "Yunnan, Baoshan, Longyang, Baihualing, on Russula sp., 20 July 2018, Jian-Wei Liu; [云南保山百花岭 2094 m, 2018.07.20, 刘建伟]; verbatimCoordinates: 25°17.931’N, 98°47.0718’E; decimalLatitude: 25.2989; decimalLongitude: 98.7845; georeferenceProtocol: label; **Identification:** identifiedBy: Feng-Ming Yu; dateIdentified: 2019

#### Description

Index Fungorum number: **IF557817**

**Figure 1.**
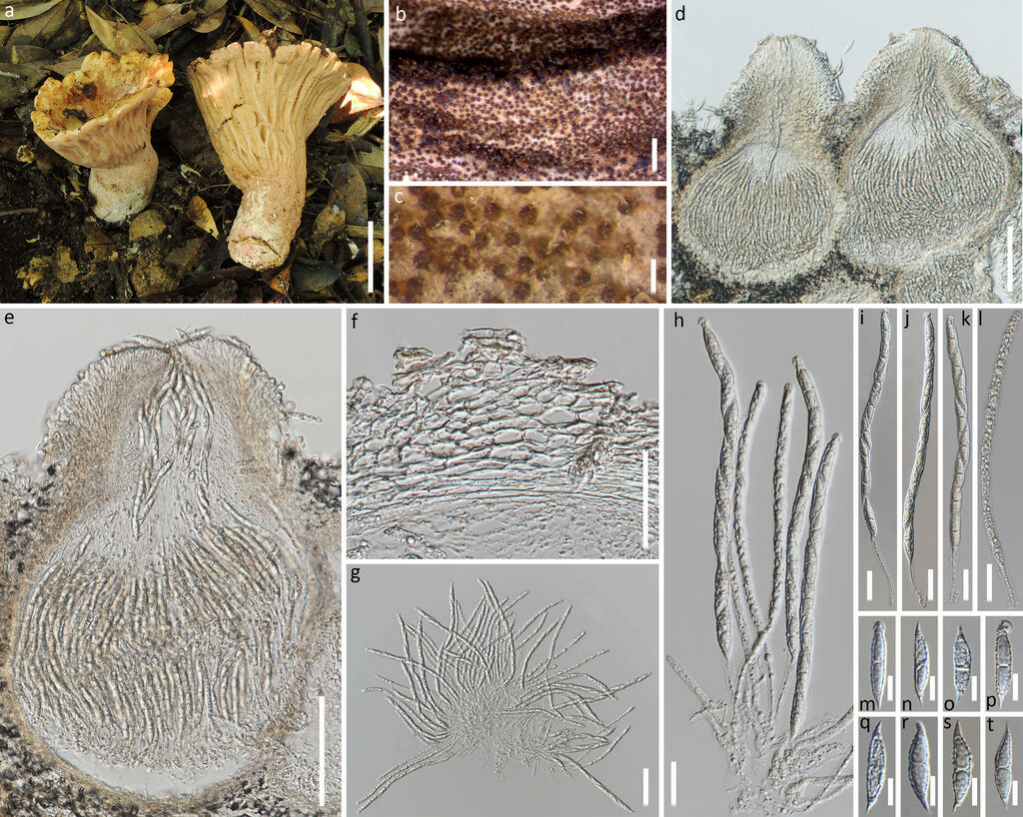
*Hypomyces pseudolactifluorum* sp. nov.. a: The host mushroom (*Russula* sp.); b-e: Perithecia embedded in subiculum effused over the substratum; d-e: Median sections of an ascoma; f: Section of peridium; g-l: Asci with ascospores; m-t: Ascospores. Scale bars: a = 5 cm; b = 1 mm; c = 200 μm; d, e = 100 μm; f, g= 50 μm; h - l = 20 μm; m - t = 10 μm.

**Sexual morph. Subiculum** light yellow (4A4–5) when fresh and pale orange, light orange to brownish-orange (5A3–4, 5C4, 6C6) after being dried, usually covering the pileus, stipe and deformed gills of the host mushroom. **Perithecia** aggregated, semi-immersed to immersed in subiculum, except for their erumpent papilla, yellowish-brown to dark brown (5E6, 6E6, 6F6–8), pyriform to subglobose, 262–484 × 136–284 μm; perithecial wall 12–25 μm thick, single-layer, cells 9–22 × 4–8 μm. **Papilla** prominent, 129–177 μm high, at base 135–284 μm wide. **Asci** 8-spored, cylindrical, 147–222 × 4–9 μm; apex thickened, 4.9–6.0 wide and 2.5-3.0 μm high. **Ascospores** uniseriate and with ends overlapping, fusiform, 30–38 × 6–8 μm, single-septate, septum median and with dense verrucae and prominently apiculate, apiculi 4.5–8.0 μm long, straight or curved. **Asexual morph**: unknown. (Fig. 1)

#### Diagnosis

The new species is similar to *Hypomyces lactifluorum* on *Russula* and *Lactarius* spp. from North America (Rogerson and Samuels 1994), but has smaller perithecia and shorter asci. The main differences of the two species are compared in Table 4.

#### Etymology

Referring to the most closely-related species, *Hypomyces lactifluorum.*

#### Distribution

PR CHINA (Yunnan).

#### Host

On the fruiting bodies of *Russula* sp. that grew on the humus layer in an evergreen broad-leaved forest of a rainforest. The host mushrooms: basidiocarps medium-sized and infundibuliform, pilei 63−77 mm in diameter. As serious degradation has occurred, the colour and other characters of the host mushrooms cannot be determined. Molecular phylogenetic evidence indicates it is a *Russula* species.

#### Notes

Only sexual morph had been discovered on the hosts (*Russula* sp.) of the new species.

## Analysis

### Phylogenetic analyses

**Parasitic fungus**: ***Hypomyces pseudolactifluorum*** **sp. nov**

**Figure 2.**
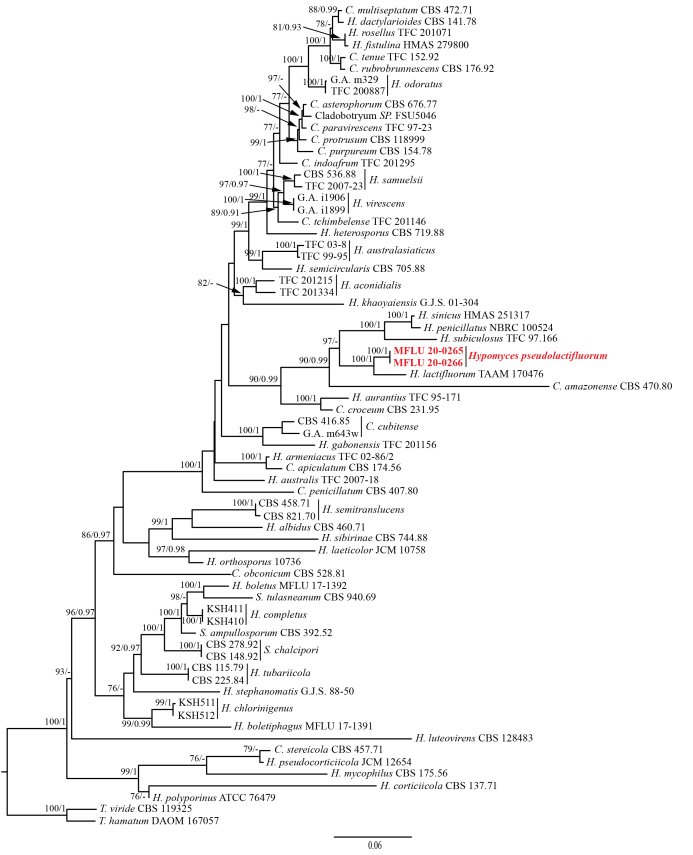
ML tree of *Hypomyces pseudolactifluorum* sp. nov. and its allies generated from a combined ITS, LSU,*TEF1-α* and RPB2 gene sequence dataset. Supporting values of MLBP (left, greater than 75%) and BIBP (right, greater than 0.9) are shown at the nodes, respectively. The new species is marked in red.

The combined ITS+LSU+*TEF1-α*+RPB2 sequence dataset (excluding the outgroup taxa) contains 3,262 characters (709 for ITS, 893 for LSU, 921 for *TEF1-α* and 739 for RPB2) from 56 *Hypomyces* species and two *Trichoderma* species. Amongst them, 2,246 characters are constant, 209 variable characters are parsimony-uninformative and 807 characters are parsimony-informative. The ML and BI analyses resulted in trees with similar topology and support values and the ML tree is shown in Fig. 2.

In the phylogenetic tree, the parasitic fungi MFLU 20-0265 and MFLU 20-0266 are clustered together and formed a distinct lineage with the same branch length and strong supportive values (MLBP = 100%, BIPP = 1), which support them to be conspecific. The parasitic fungi are closely related *H. lactifluorum* and they form a sister clade also with strong supportive values (MLBP = 100%, BIPP = 1). Comparing the gene sequences of the two species, there are 25 bp (4.3%) differences across 582 bp in ITS, 28 bp (3.2%) differences across 870 bp in LSU, 24 bp (2.6%) differences across 921 bp in *TEF1-α* and 24 bp (3.2%) differences across 739 bp in RPB2 (Suppl. material 1). Following the recommendations from Jeewon and Hyde (2016), we assign the parasitic fungi as *H. pseudolactifluorum* sp. nov.

**The host mushroom: *Russula* sp.**

**Figure 3.**
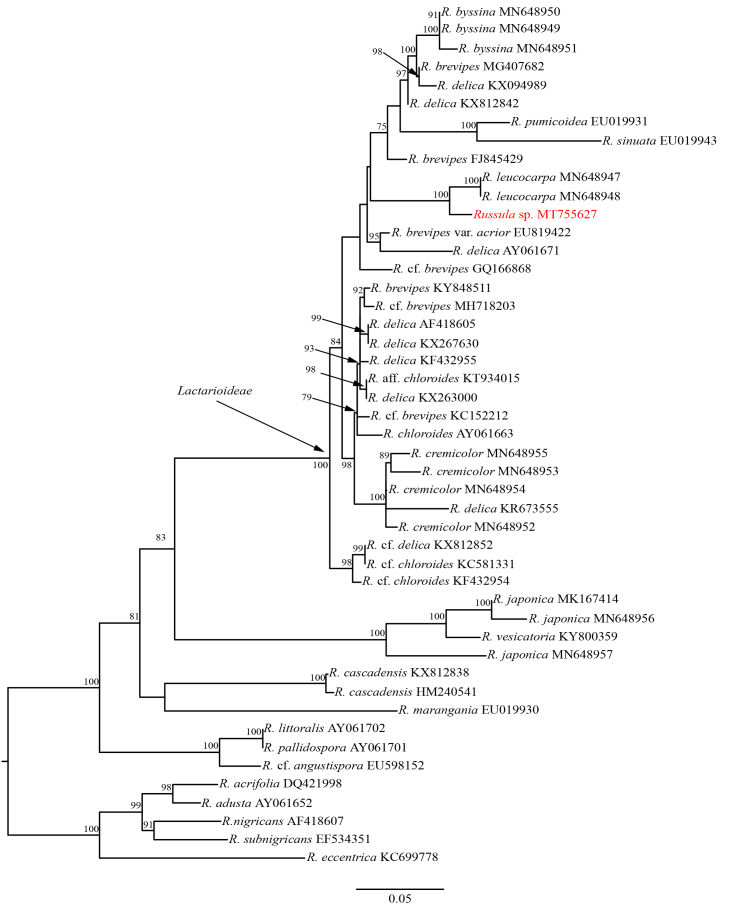
ML tree of *Russula* sp. (in red) and it allies inferred from the ITS sequence dataset. Five species of Russula subg. Compactae were used as the outgroup taxa. Supporting values of MLBP (greater than 75%) are shown at the nodes.

According to the ITS phylogenetic tree of the host mushroom and its allies, the host mushroom (MFLU 20-0265) is clustered together with *Russula leucocarpa* (HGAS-MF 009910 and HGAS-MF 009916) (MLBP = 100%) in subsect. Lactarioideae. However, their ITS sequences have 24 bp (3.5%) differences across 694 bp, which indicated they may be two distinct species. Due to lack of sufficient morphological evidence, the host mushroom was temporarily identified as *Russula* sp. (Fig. 3).

## Discussion

Zeng and Zhuang (2016) described *H. amaniticola* on *Amanita* sp. and *H. completiopsis* and *H. yunnanensis* on *Boletus* sp., also from China. Though with similar colour and shapes of perithecia, the host of *H. pseudolactifluorum* sp. nov. is decidedly different from those of these three species. Furthermore, *H. pseudolactifluorum* sp. nov. (KOH-) has smaller perithecia and larger ascospores than those of *H. completiopsis* (KOH^+^) and *H. pseudolactifluorum* sp. nov. has larger perithecia, asci and ascospores than those of *H. amaniticola* (KOH^+^) and *H. yunnanensis* (KOH-). Unfortunately, these three species all lack molecular data.

With the rapid development of mushroom industries, fungal pathogens on mushrooms have received more and more attention (Hyde et al. 2019). The fungicolous fungi *Hypomyces* is an important group of mushroom pathogens. Many *Hypomyces* species, for example, *H. aurantius*, *H. perniciosus*, *H. rosellus*, *H. odoratus* etc., have all been recorded as the causes of Cobweb or Web bubble disease which seriously influence mushroom industries (Fletcher and Gaze 2007, Carrasco et al. 2017, Zhang et al. 2017, Zhang et al. 2017). Russula is the largest subgenus in agaric with approximately 800 species (Li et al. 2020) and many *Russula* species are important edible mushrooms. Since growing on *Russula* sp., *H. pseudolactifluorum* sp. nov., as well as *H. lactifluorum* from North America (Rogerson and Samuels 1994), could be one of the potential pathogens of some *Russula* species in Asia.

## Supplementary Material

168B0805-53B9-5CD4-BC29-6578A8F13263 10.3897/BDJ.8.e53490.suppl1 Supplementary material 1 *Hypomyces pseudolactifluorum* sp. nov. (Hypocreales: Hypocreaceae) on *Russula* sp. from Yunnan, PR China Data type word Brief description Sequence differences of ITS, LSU, *TEF1*-*α* and RPB2 genes between *H. lactifluorum* (TAAM 170476) and *H. pseudolactifluorum* sp. nov.. The locus’ numbers refer to the nucleotide positions of the gene sequences of *H. lactifluorum* from GenBank. Gap is replaced by ‘-’. File: oo_438866.docx https://binary.pensoft.net/file/438866 FENG-MING YU, RUVISHIKA S. JAYAWARDENA, JIAN-WEI LIU, KEVIN D. HYDE, QI ZHAO

XML Treatment for Hypomyces pseudolactifluorum

## Figures and Tables

**Table 1.** Species diversity of the genus *Hypomyces* in China (29 species in total).

**Taxa names**	**Hosts**	**Distribution**	**References**
*Hypomyces amaniticola*	*Amanita* sp.	China (Yunnan)	Zeng and Zhuang 2016
*H. aurantius*	*Agaricus bisporus*, Polyporales (*Cymatoderma* sp., *Laetiporus sulphureus*, *Panellus* sp., *Polyporus picipes*), *Stereum* sp.	China (Anhui, Fujian, Guangxi, Hainan, Hebei, Hunan, Jiangsu, Jiangxi, Shanghai, Sichuan, Zhejiang), New Zealand, USA	Chen and Fu 1989, Põldmaa 2011, Luo and Zhuang 2012
*H. aureonitens*	*Phlebia tremellosa*, *Polyporus* sp.	China (Fujian, Guangxi), Europe	Teng 1963, Sun et al. 2019a
*H. chlorinigenus*	Agaricaceae, Boletaceae	Belgium, China (Taiwan), Guyana; Indonesia, New Zealand, USA	Rogerson and Samuels 1989, Zeng and Zhuang 2016
*H. chrysospermus*	*Boletus* sp., *Hemileccinum impolitum*, *Suillus americanus*, *Russula* sp.	China (Fujian, Jiling, Nanjing), Russia	Ma 2008, Luo and Zhuang 2012
*H. completiopsis*	*Boletus* sp.	China (Yunnan)	Zeng and Zhuang 2016
*H. fistulina*	*Fistulina* sp.	China (Guangxi)	Sun et al. 2019b
*H. hubeiensis*	*Agaricus* sp.	China (Hubei)	Zeng and Zhuang 2019
*H. hyalinus*	Agaricales (*Amanita* sp.), Polyporales	Canada, China (Jiangsu), Japan, USA	Teng 1934, Teng 1963, Rogerson and Samuels 1994
*H. lateritius*	*Lactarius camphoratus*, *L. chelidonium*, *L. controversus*, *L. deliciosus*, *L. sanguifluus*, *L. thejogalus*, *L. trivialis*, *Lactarius* sp.	Canada, China (Tibet), Europe, Japan, Mexico, New Zealand, USA	Rogerson and Samuels 1994, Luo and Zhuang 2012
*H. luteovirens*	*Russula atropurpurea*, *R. rosea*, *R. sanguinaria*, *Russula* sp.	Canada, China (Inner Mongolia), Europe, Japan, Russia, USA	Rogerson and Samuels 1994, Ma 2008
*H. macrosporus*	Russulaceae	China (Hubei), Mexico, USA	Rogerson and Samuels 1994, Luo and Zhuang 2012
*H. microspermus*	Boletaceae, *Boletus* sp., *Imleria badia*, *Xanthoconium affine*, *Xerocomellus chrysenteron*, *Xerocomus* sp.	Canada, China (Fujian, Guizhou, Hainan, Hubei, Jilin, Taiwan, Yunan), Indonesia, New Zealand, USA	Rogerson and Samuels 1989, Zeng and Zhuang 2016
*H. mycophilus*	*Auricularia* sp., *Bulgari* sp., *Marasmius* sp., *Polyporus* sp., *Trametes versicolor*	China (Guangdong), USA	Rogerson and Samuels 1993, Zeng et al. 2017
*H. ochraceus*	Decaying leaves, wood and fungi (e.g. *Russula* sp.)	China (Guangxi, Yunnan), Europe, USA	Teng 1963, Sun et al. 2019a
*H. orthosporus*	Polyporales	China (Tibet), Estonia, Finland, The Netherlands	Põldmaa 1996, Zeng and Zhuang 2019
*H. papulasporae*	*Geoglossum difforme*, *G. fallax*, *G. glabrum*, *G. nigritum*, *G. simile*, *Glutinoglossum glutinosum*, *Trichoglossum hirsutum*, *T. walteri*	China, USA, New Zealand	Rogerson and Samuels 1985, Sun et al. 2019a
*H. polyporinus*	*Auricularia auricula-judae*, Polyporales, *Trametes versicolor*, *T. pubescens*, *Polyporus* sp.	Canada, China (Guangxi), USA	Teng 1963, Rogerson and Samuels 1993
***H. pseudolactifluorum* sp. nov.**	***Russula* sp.**	**China (Yunnan)**	**This study**
*H. rosellus*	*Agaricus bisporus*, *Armillaria* sp., *Hydnellum* sp., *Hyphoderma* sp., *Mycena* sp., *Polyporus* sp., *Russula* sp., *Trichaptum* sp.	China (Gansu), Europe, Iran, Japan, Korea, USA	Tamm and Põldmaa 2013, Sun et al. 2019b
*H. semicircularis*	*Ganoderma sichuanense*, *Microporus xanthopus*	Cuba, China	Wei and Kirschner 2017, Sun et al. 2019a
*H. sibirinae*	Aphyllophorales, *Boletus* sp., Polyporales	China (Hunan), Indonesia, USA	Samuels et al. 1990, Zeng et al. 2017, Sun et al. 2019a
*H. sinicus*	*Schizophyllum* sp.	China (Anhui)	Zhuang et al. 2012
*H. stephanomatis*	*Humaria hemisphaerica*, *Humaria* sp.	Canada, China (Hubei), Germany, USA	Rogerson and Samuels 1985,Zeng and Zhuang 2016
*H. subiculosus*	Polyporaceae (*Microporus affinis*, *Trametes versicolo*)	China (Anhui, Beijing, Guangxi, Zhejiang), Cuba, Japan	Rogerson and Samuels 1993, Luo and Zhuang 2012
*H. succineus*	*Pholiota* sp.	China (Taiwan), USA	Rogerson and Samuels 1994, Zeng and Zhuang 2016
*H. tegillum*	Aphyllophorales, Polyporales	Brazil, China (Guangxi, Yunnan), Panama, USA	Rogerson and Samuels 1993, Luo and Zhuang 2012
*H. triseptatus*	Bark or associated with an ascomycete; Pyrenomycete	China (Hunan, Guangdong), Gabon	Rossman and Rogersson 1981, Zeng et al. 2017
*H. yunnanensis*	*Boletus* sp.	China (Yunnan)	Zeng and Zhuang 2016

**Table 2.** Voucher information and GenBank accession numbers for samples appearing in the *Hypomyces* phylogenetic tree. Our sequencing results are displayed in bold. (Label T indicate the sequences from ex-type strains.)

**Taxa names**	**Specimen/Strain** **number**	**GenBank accession numbers**				**References**
		**ITS**	**LSU**	***TEF1-α***	**RPB2**	
*Cladobotryum amazonense*	CBS 470.80	MH861285	MH873051	/	/	Vu et al. 2019
*C. apiculatum*	CBS 174.56 ^T^	NR_159770	MH869109	/	/	Vu et al. 2019
*C. asterophorum*	CBS 676.77 ^T^	FN859395	MH872869	FN868712	FN868649	Põldmaa 2011
*C. croceum*	CBS 231.95	MH862511	MH874154	/	/	Vu et al. 2019
*C. cubitense*	CBS 416.85	FN859396	/	FN868713	FN868650	Põldmaa 2011
	G.A. m643.w	FN859397	/	FN868714	FN868651	Põldmaa 2011
*C. indoafrum*	TFC 201295	FN859403	FN859403	FN868721	FN868657	Põldmaa 2011
*C. multiseptatum*	CBS 472.71 ^T^	FN859405	MH871991	FN868723	FN868659	Põldmaa 2011
*C. obconicum*	CBS 528.81	MH861373	MH873126	/	/	Vu et al. 2019
*C. paravirescens*	TFC 97-23 ^T^	FN859406	FN859406	FN868724	FN868660	Põldmaa 2011
*C. penicillatum*	CBS 407.80 ^T^	FN859407	MH873046	FN868725	FN868661	Põldmaa 2011
*C. protrusum*	CBS 118999	FN859408	FN859408	FN868726	FN868662	Põldmaa 2011
*C. purpureum*	CBS 154.78 ^T^	FN859415	/	FN868733	FN868669	Põldmaa 2011
*C. rubrobrunnescens*	CBS 176.92 ^T^	FN859416	MH874016	FN868734	FN868670	Põldmaa 2011
*Cladobotryum* sp.	FSU 5046	FN859421	/	FN868739	FN868675	Põldmaa 2011
*C. stereicola*	CBS 457.71 ^T^	MH860217	MH871984	/	/	Vu et al. 2019
*C. tchimbelense*	TFC 201146 ^T^	FN859419	FN859419	FN868737	FN868673	Põldmaa 2011
*C. tenue*	CBS 152.92 ^T^	FN859420	FN859420	FN868738	FN868674	Põldmaa 2011
*Hypomyces aconidialis*	TFC 201334 ^T^	FN859457	FN859457	FN868775	FN868711	Põldmaa 2011
	TFC 201215	FN859456	FN859456	FN868774	FN868710	Põldmaa 2011
*H. albidus*	CBS 460.71	MH860220	MH871987	/	/	Vu et al. 2019
*H. armeniacus*	TFC 02-86/2 ^T^	FN859424	FN859424	FN868742	FN868678	Põldmaa 2011
*H. aurantius*	TFC 95-171	FN859425	FN859425	FN868743	FN868679	Põldmaa 2011
*H. australasiaticus*	TFC 03-8 ^T^	FN859428	FN859428	FN868746	FN868681	Põldmaa 2011
	TFC 99-95	FN859427	/	FN868745	FN868680	Põldmaa 2011
*H. australis*	TFC 2007-18	AM779860	AM779860	FN868747	/	Põldmaa 2011
*H. boletiphagus*	MFLU 17-1391	MH459152	MH459168	/	MH464785	Sun et al. 2019b
*H. boletus*	MFLU 17-1392	MH459153	MH459170	/	MH464787	Sun et al. 2019b
*H. chlorinigenus*	KSH511	KT946843	/	KU041505	KU041493	Otto et al. 2016
	KSH512 ^T^	KT946844	/	KU041506	KU041494	Otto et al. 2016
*H. completus*	KSH411 (S172)	KT946842	/	KU041504	KU041492	Otto et al. 2016
	KSH410 (S171) ^T^	KT946841	/	KU041503	KU041491	Otto et al. 2016
*H. corticiicola*	CBS 137.71 ^T^	MH860037	MH871817	/	/	Vu et al. 2019
*H. dactylarioides*	CBS 141.78 ^T^	FN859429	MH872879	FN868748	FN868683	Põldmaa 2011
*H. fistulina*	HMAS 279800 ^T^	MH459154	MH459171	MH464781	/	Sun et al. 2019b
*H. gabonensis*	TFC 201156 ^T^	FN859430	FN859430	FN868749	FN868684	Põldmaa 2011
*H. heterosporus*	CBS 719.88 ^T^	FN859398	MH873844	FN868716	FN868653	Põldmaa 2011
*H. khaoyaiensis*	G.J.S. 01-304 ^T^	FN859431	AJ583483	FN868750	FN868685	Põldmaa 2011
*H. lactifluorum*	TAAM 170476 ^T^	FN859432	EU710768	FN868751	EU710773	Põldmaa 2011
*H. laeticolor*	JCM 10758 ^T^	LC228655	LC228712	/	/	Sun et al. 2019b
*H. luteovirens*	CBS 128483	MH864958	MH876402	/	/	Vu et al. 2019
*H. mycophilus*	CBS 175.56	MH857567	MH869110	/	/	Vu et al. 2019
*H. odoratus*	G.A. m329	FN859434	FN859434	FN868753	FN868688	Põldmaa 2011
	TFC 200887	FN859439	/	FN868757	FN868693	Põldmaa 2011
*H. orthosporus*	10736	MK478468	MN044763	MK484609	/	Zeng and Zhuang 2019
*H. penicillatus*	NBRC 100524	LC146740	LC146740	/	/	Sun et al. 2019b
***H. pseudolactifluorum* sp.nov.**	**MFLU 20-0265** ^T^	**MT260402**	**MT260399**	**MT259361**	**MT259359**	**This study**
	**MFLU 20-0266**	**MT260403**	**MT260400**	**MT259362**	**MT259360**	**This study**
*H. polyporinus*	ATCC 76479	AF543771	AF543793	AF543784	/	Currie et al. 2003
*H. pseudocorticiicola*	JCM 12654 ^T^	LC228663	LC228721	/	/	Sun et al. 2019b
*H. rosellus*	TFC 201071	FN859443	FN859443	FN868762	FN868697	Põldmaa 2011
*H. samuelsii*	CBS 536.88	FN859444	/	FN868763	FN868698	Põldmaa 2011
	TFC 2007-23	FN859451	FN859451	FN868769	FN868705	Põldmaa 2011
*H. semicircularis*	CBS 705.88 ^T^	FN859417	MH873843	FN868735	FN868671	Põldmaa 2011
*H. semitranslucens*	CBS 458.71	MH860218	MH871985	/	/	Vu et al. 2019
	CBS 821.70	MH859960	MH871759	/	/	Vu et al. 2019
*H. sibirinae*	CBS 744.88	MH862151	AJ459304	/	/	Vu et al. 2019
*H. sinicus*	HMAS 251317 ^T^	NR_156252	MN044986	MK484610	/	Zhuang et al. 2012
*H. stephanomatis*	G.J.S. 88-50	/	AF160243	AF534632	AF545566	Põldmaa et al. 2000
*H. subiculosus*	TFC 97.166	FN859452	/	FN868770	FN868706	Põldmaa 2011
*H. tubariicola*	CBS 115.79 ^T^	KU382164	MH872953	/	/	Vu et al. 2019
	CBS 225.84	KU382162	KU382220	/	/	Zare and Gams 2016
*H. virescens*	G.A. i1906 ^T^	FN859454	/	FN868772	FN868708	Põldmaa 2011
	G.A. i1899	FN859453	/	FN868771	FN868707	Põldmaa 2011
*Sepedonium ampullosporum*	CBS 392.52 ^T^	MH857094	MH868629	/	/	Vu et al. 2019
*S. chalcipori*	CBS 278.92	MH862358	MH874023	/	/	Vu et al. 2019
	CBS 148.92 ^T^	MH862347	MH874014	/	/	Vu et al. 2019
*S. tulasneanum*	CBS 940.69	MH859489	MH871270	/	/	Vu et al. 2019
*Trichoderma hamatum*	DAOM 167057 ^T^	EU280124	HM466686	AF534620	AF545548	Hoyos-Carvajal et al. 2009
*T. viride*	CBS 119325 ^T^	DQ677655	/	DQ672615	EU711362	Jaklitsch et al. 2006

**Table 3.** Voucher information and GenBank accession numbers for samples appearing in the *Russula* phylogenetic tree. Our sequencing results are displayed in bold.

**Taxa names**	**Specimen/Strain number**	**GenBank accession**	**References**
*Russula acrifolia*	TUB UE12.09.2003-3	DQ421998	Eberhardt 2002
*R. adusta*	PC 547RUS27	AY061652	Miller and Buyck 2002
R. aff. chloroides	FH 12273	KT934015	Looney et al. 2016
*R. brevipes*	SMI329	FJ845429	Kranabetter et al. 2009
*R. brevipes*	JS160927-01	MG407682	GenBank
*R. brevipes*	TENN 070667	KY848511	Looney et al. 2018
R. brevipes var. acrior	JMP 0058	EU819422	Palmer et al. 2008
*R. byssina*	HGAS-MF 009907	MN648951	Li et al. 2020
*R. byssina*	HGAS-MF009921	MN648949	Li et al. 2020
*R. byssina*	HGAS-MF 009913	MN648950	Li et al. 2020
*R. cascadensis*	UBC F30189	KX812838	Bazzicalupo 2018
*R. cascadensis*	UBC F19691	HM240541	Buyck et al. 2017
R. cf. angustispora	PC BB2004-252	EU598152	GenBank
R. cf. brevipes	F 28785	MH718203	GenBank
R. cf. brevipes	F CDW47	GQ166868	GenBank
R. cf. brevipes	GO 2009-276	KC152212	GenBank
R. cf. delica	UBC F30260	KX812852	Bazzicalupo 2018
*R. chloroides*	PC 205RUS24	AY061663	Miller and Buyck 2002
*R. chloroides*	UBC F20353	KC581331	GenBank
*R. chloroides*	RUS-12091401	KF432954	Wisitrassameewong et al. 2014
*R. cremicolor*	HGAS-MF 009901	MN648955	Li et al. 2020
*R. cremicolor*	HGAS-MF 009908	MN648952	Li et al. 2020
*R. cremicolor*	HGAS-MF 009912	MN648953	Li et al. 2020
*R. cremicolor*	HGAS-MF 009919	MN648954	Li et al. 2020
*R. delica*	hue22 (TUB)	AF418605	Eberhardt 2002
*R. delica*	FH 12-272	KF432955	Wisitrassameewong et al. 2014
*R. delica*	HA 2015-004	KX263000	Aghajani et al. 2017
*R. delica*	PC 496RUS26	AY061671	Miller and Buyck 2002
*R. delica*	TUB hue22	AF418605	Eberhardt 2002
*R. delica*	UBC F30263	KX812842	Bazzicalupo 2018
*R. delica*	RMUKK 37	KX267630	GenBank
*R. delica*	KA 12-1327	KR673555	Kim et al. 2015
*R. delica*	HMJAU 32182	KX094989	Liu et al. 2017
*R. eccentrica*	HCCN 23685	KC699778	Park et al. 2014
*R. japonica*	MHHNU 31049	MK167414	Chen and Zhang 2019
*R. japonica*	HGAS-MF 009923	MN648957	Li et al. 2020
*R. japonica*	HGAS-MF 009915	MN648956	Li et al. 2020
*R. leucocarpa*	HGAS-MF 009910	MN648948	Li et al. 2020
*R. leucocarpa*	HGAS-MF 009916	MN648947	Li et al. 2020
*R. littoralis*	PC 1222IS87	AY061702	Miller and Buyck 2002
*R. marangania*	MEL 2293694	EU019930	Lebel and Tonkin 2007
*R. nigricans*	TUB fo46761	AF418607	Eberhardt 2002
*R. pallidospora*	PC 2-1221IS85	AY061701	Miller and Buyck 2002
*R. pumicoidea*	MEL T-14771	EU019931	Lebel and Tonkin 2007
*R. sinuata*	MEL H4755	EU019943	Lebel and Tonkin 2007
*R. subnigricans*	MHHNU ZP6932	EF534351	Yin et al. 2008
*R. vesicatoria*	PC 0124666	KY800359	Buyck et al. 2017
***Russula* sp.**	**MFLU 20-0265 (host)**	**MT755627**	**In this study**

**Table 4.** Main differences between *Hypomyces lactifluorum* and *H. pseudolactifluorum* sp. nov..

	*H. lactifluorum* (Rogerson and Samuels 1994)	*H. pseudolactifluorum*
Subiculum	Pale yellowish-orange to bright orange (young), in age becoming deep red, reddish-purple to very dark purple (old), occasionally fading to pink, turning purple in 3% KOH.	Light yellow (4A4–5) when fresh, and pale orange to light orange to brownish-orange (5A3–4, 5C4, 6C6) after being dried, KOH (-).
Perithecia	Ovate to obpyriform, deep orange to reddish-purple, 400–600 × 200–450 μm	Pyriform to subglobose, yellowish-brown to dark brown (5E6, 6E6, 6F6–8), 262–484 × 136–284 μm
Embedded type	Immersed except for papilla	Semi-immersed to immersed except for papilla
Papilla	Averaging 120 μm high, 120 μm wide	129–177 μm high and 135 –284 μm wide at base
Asci	Long cylindrical, 200–260 × 5–10 μm	Cylindrical, 147–222 × 4–8.5 μm
Ascospores	Fusiform, 1-septate, 35–40 × 4.5–7 μm	Fusiform, 1-septate, 30–38 × 5.5–8 μm
Apiculi	4.5–7.5 μm long	4–6 μm long
Hosts	*Russula* and *Lactarius* spp.	*Russula* sp.
Distribution	North America	P.R. China (Yunnan)
