# Supplementary material for: Hypomyces pseudolactifluorum sp. nov. (Hypocreales: Hypocreaceae) on Russula sp. from Yunnan, PR China
Source: Biodivers Data J. 2020 Sep 28;8:e53490. doi: 10.3897/BDJ.8.e53490 (PMC7536245; doi:10.3897/BDJ.8.e53490)
Supplement: Supplementary material 1 — Hypomyces pseudolactifluorum sp. nov. (Hypocreales: Hypocreaceae) on Russula sp. from Yunnan, PR China [file bdj-08-e53490-s001.docx]

Suppl. Material 1 Sequence differences of ITS, LSU, *TEF1*-*α* and RPB2 genes between *H. lactifluorum* (TAAM 170476) and *H. pseudolactifluorum* sp. nov.. The locus’s numbers refer to the nucleotide positions of the gene sequences of *H. lactifluorum* from GenBank. Gap is replaced by ‘-’.

| Gene | ITS | | | | | | | | | | | | | | | | | | | | | | | | | Ratio |
| --- | --- | --- | --- | --- | --- | --- | --- | --- | --- | --- | --- | --- | --- | --- | --- | --- | --- | --- | --- | --- | --- | --- | --- | --- | --- | --- |
| The locus | 19 | 29 | 117 | 118 | 131 | 147 | 153 | 156 | 160 | 166 | 196 | 275 | 296 | 351 | 363 | 372 | 392 | 430 | 450 | 506 | 518 | 523 | 524 | 525 | 531 | 4.3%  (25/582) |
| *H. lactifluorum* | A | C | A | A | G | A | - | T | A | - | G | T | T | C | T | - | G | A | A | T | G | - | - | - | A |  |
| *H. pseudolactifluorum* | G | T | G | T | A | G | A | C | G | C | - | C | C | T | C | C | C | T | G | C | A | C | C | C | C |  |
| Gene | LSU | | | | | | | | | | | | | | | | | | | | | | | | |  |
| Locus | 63-68 | | 69 | 70 | 93 | 108 | 112 | 113 | 167 | 389 | 406 | 409 | 434 | 465 | 495 | 507 | 515 | 517 | 595 | 617 | 632 | 658 | 687 | 816 |  | 3.2%  (28/870) |
| *H. lactifluorum* | C | | G | G | A | R | S | S | C | T | T | G | G | T | T | T | G | C | C | T | C | C | C | G |  |  |
| *H. pseudolactifluorum* | - | | - | - | G | G | C | G | T | C | C | A | A | C | C | C | A | T | T | C | T | T | T | A |  |  |
| Gene | *TEF1*-*á* | | | | | | | | | | | | | | | | | | | | | | | | |  |
| Locus | 66 | 147 | 273 | 372 | 375 | 381 | 435 | 498 | 566 | 585 | 591 | 594 | 627 | 666 | 670 | 671 | 675 | 687 | 762 | 833 | 867 | 903 | 907 | 909 |  | 2.6%  (24/921) |
| *H. lactifluorum* | T | C | C | C | G | T | T | C | A | T | T | C | T | C | A | C | A | T | C | C | T | C | T | T |  |  |
| *H. pseudolactifluorum* | C | A | T | T | A | C | C | T | C | C | C | T | C | T | G | T | T | C | T | T | C | T | G | C |  |  |
| Gene | RPB2 | | | | | | | | | | | | | | | | | | | | | | | | |  |
| Locus | 3 | 15 | 60 | 110 | 126 | 234 | 240 | 255 | 294 | 393 | 399 | 420 | 450 | 528 | 543 | 548 | 561 | 582 | 639 | 654 | 672 | 678 | 707 | 735 |  | 3.3%  (24/739) |
| *H. lactifluorum* | A | C | T | A | A | T | T | A | A | T | G | T | T | A | C | G | C | A | T | A | C | A | G | A |  |  |
| *H. pseudolactifluorum* | T | T | C | C | C | C | C | G | G | C | A | C | C | G | T | A | T | G | C | C | T | G | A | T |  |  |
